# Correction: The Role of Heat Shock Proteins in Cellular Homeostasis and Cell Survival

**DOI:** 10.7759/cureus.c52

**Published:** 2021-11-09

**Authors:** Abdullah Farhan Y Almalki, Maria Arabdin, Adnan Khan

**Affiliations:** 1 Pathology, University of Malta, Msida, MLT; 2 Pathology, Rehman Medical College, Peshawar, PAK; 3 Pediatrics, Rehman Medical Institute, Peshawar, PAK

Figure 3 was originally published in Expert Reviews in Molecular Medicine (2001) Cambridge University Press. This information was mistakenly deleted from the figure legend during revisions and has now been re-added to the Figure 3 legend. Cureus regrets the error.

